# Metagenomic profile of gut microbiota in children during cholera and recovery

**DOI:** 10.1186/1757-4749-5-1

**Published:** 2013-02-01

**Authors:** Shirajum Monira, Shota Nakamura, Kazuyoshi Gotoh, Kaori Izutsu, Haruo Watanabe, Nur Haque Alam, Takaaki Nakaya, Toshihiro Horii, Sk Imran Ali, Tetsuya Iida, Munirul Alam

**Affiliations:** 1 International Center for Diarrhoeal Disease Research, Bangladesh; 2 Research Institute for Microbial Diseases, Osaka University, 3-1 Yamadaoka, Suita, Osaka, 565-0871, Japan; 3 National Institute of Infectious Diseases, Shinjuku-ku, Tokyo, Japan; 4 Department of Infectious Diseases, Kyoto Prefectural University of Medicine, 465 Kawaramachi-hirokoji, Kamigyo-ku, Kyoto, Japan

**Keywords:** Cholera, Microbiota, Gut, 16S rDNA, Children

## Abstract

**Background:**

The diverse bacterial communities colonizing the gut (gastrointestinal tract) of infants as commensal flora, which play an important role in nutrient absorption and determining the state of health, are known to alter due to diarrhea.

**Method:**

Bacterial community dynamics in children suffering from cholera and during recovery period were examined in the present study by employing metagenomic tool, followed by DNA sequencing and analysis. For this, bacterial community DNA was extracted from fecal samples of nine clinically confirmed cholera children (age 2–3 years) at day 0 (acute cholera), day 2 (antibiotic therapy), day 7 and, and day 28, and the variable region of 16S rRNA genes were amplified by universal primer PCR.

**Results:**

454 parallel sequencing of the amplified DNA followed by similarity search of the sequenced data against an rRNA database allowed us to identify *V. cholerae*, the cause of cholera, in all nine children at day 0, and as predominant species in six children, accounting for 35% of the total gut microbiota on an average in all the nine children. The relative abundance (mean ± sem %) of bacteria belonging to phyla Proteobacteria, Firmicutes, Bacteroidetes, and Actinobacteria, was 55 ± 7, 18 ± 4, 13 ± 4, and 8 ± 4, respectively, at day 0, while these values were 12 ± 4, 43 ± 4, 33 ± 3, and 12 ± 2, respectively, at day 28. As antibiotic therapy began, *V. cholerae* count declined significantly (p< 0.001) and was found only in four children at day 2 and two children at day 7 with the relative abundance of 3.7% and 0.01%, respectively, which continued up to day 28 in the two children. Compared to acute cholera condition (day 0), the relative abundance of *Escherichia coli, Enterococcus,* and *Veillonella* increased at day 2 (antibiotic therapy) while *Bifidobacterium, Bacteroides,* and *Ruminococcus* decreased.

**Conclusion:**

Cholera results expulsion of major commensal bacteria of phyla Bacteroidetes, Firmicutes, and Actinobacteria, and increase of harmful Proteobacteria to colonize the gut during acute and convalescence states. The observed microbiota disruption might explain the prevalent malnutrition in children of Bangladesh where diarrheal diseases are endemic.

## Background

The human gut is a complex ecosystem with a huge number of microorganisms, collectively known as microbiota. The gut microbiota consists of a diverse population of prokaryotic (eubacteria and archaea) as well as eukaryotic microbes that live synergistically within their human host and called commensal microbiota [1]. The adult gastrointestinal tract acquires at least 17 families of bacteria yielding 400 to 500 different microbial species with regional variation of bacterial composition within the gastrointestinal tract. In general, there is a qualitative and quantitative increase in complexity from the stomach to the colon. These commensal bacteria regulate a number of host processes from nutrition and development to immune responses functionally regulating both health and disease [2,3].

Fecal microbiota obtained from the cholera children on different time periods e.g., during acute cholera, convalescence and recovery was analyzed using culture independent molecular techniques in a previous study in Laboratory Sciences Division, icddr,b [4]. The general aim of the study was to measure the quantity and biodiversity of colonic microbiota as a function of time in children with cholera by a community fingerprint technique called temporal temperature gradient gel electrophoresis (TTGE). PCR-TTGE analysis of dominant bacteria from 16S rDNA of seven fecal samples of each patient revealed various profiles comprising of four to seventeen bands. In a first approximation and for the sake of communication, we assumed that each band corresponded to a different bacterial species. In acute cholera condition, a reduced number of bands were observed which started to increase steadily from day 2 to day 7. Although we came to know about diversity pattern during disease and convalescent phase we are unaware of dominant bacterial species during these periods. Many factors such as age, geographic locations, diets, pH, bile acid, and most important the intestinal infections determine the nature and composition of resident bacterial populations in the colon [5]). In acute cholera, the gut of patients is washed out due to purging and frequent loss of loose stools. This also causes the anaerobic environment to be disrupted and the commensal microbiota to expel out and replaced by aerobic and pathogenic bacteria. Previous studies have examined the gastrointestinal microbiota in acute cholera [6,7] and in acute diarrhoea cases [8,9] using conventional culture techniques, microscopy, and biochemical tests, showing that the faecal anaerobic bacteria were significantly reduced in number during acute disease leading to aerobic bacteria to increase several folds. The major anaerobic bacterial group, including *Bacteroides, Clostridium, Bifidobacterium, Lactobacillus,* and *Eubacterium,* was found 3–4 times lower in acute cholera and diarrhoea patients. The designing of an appropriate therapeutic intervention intended to use the metabolism of intestinal microbiota for rapid recovery of children from cholera and achieve regular status of gut microbiota requires a clear understanding of its composition and functions. The aim of this study was to assess the major bacterial phyla in children using an un-biased high-throughput sequencing approach, the pyrosequencing to determine the extent of loss of gut bacteria during acute and convalescence period of cholera.

## Results

### Study subjects

The study subjects (n=9) included in this study were all children aged between 2–3 years. For all the study children, who were suffering from acute dehydrating diarrhea primarily confirmed as cholera, the average duration for cessation of cholera was 72 hours. The hospital stays for these cholera patients were variable, ranging from 5 – 7 days.

### Universal primer PCR and pyrosequencing

To determine the bacterial lineages present in the fecal microbiota of children with cholera, pyrosequencing technology was adopted targeting the PCR-amplified hyper variable region, V5-V6, of the 16S rRNA gene with a GS Junior platform (454 Life Sciences). The sequencing generated a dataset consisting of filtered high quality classifiable 16S rRNA gene sequences with a mean ± SD of 13,966 ± 7,297 sequences per sample.

### Pre- and post-antibiotic pattern of *major* bacteria in cholera

**Figure 1:**
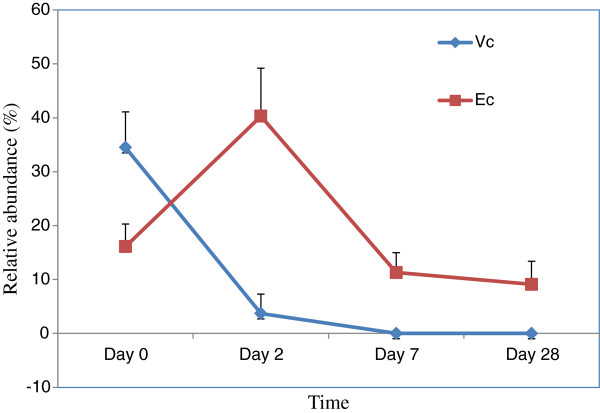
**Relative abundance (percentage) of the sequences showing the best hit to *V. cholerae *(Vc) and *Escherichia coli *(Ec) on different time points in children with cholera.** The data is relative abundance in total gut microbiota of all the patients.

At day 0, before administration of any antibiotic, cholera was confirmed in all nine children by the presence of *Vibrio cholerae* sequences. The overall relative abundance of *V. cholerae* sequences was determined to be 35% of the total gut microbiota of the patients (Figure 1). The highest and the lowest relative abundances were 63% and 5%, respectively, in this group of children with cholera. After the commencement of antibiotic therapy, *V. cholerae* abundance declined significantly (p< 0.001) and the bacterium was found only in four children at day 2 with the relative abundance (RA) of 3.7%, and in two children at day 7 with RA of 0.01%. Interestingly, the two children that had *V. cholerae* in their gut at day 7 maintained the bacterium for up to day 28, with the RA of 0.01%. As the RA of *V. cholerae* declined sharply following the initiation of antibiotic therapy, the abundance of sequences showing the best hit to *Escherichia coli* increased sharply and became the predominant with the RA of 40% in total gut microbiota of nine cholera children (Figure 1). This antibiotic-guarded floral pattern changed again as the antibiotic therapy ended. As a corollary, the abundance of *E. coli* declined sharply at day 7 with the RA of 11.3%. Since then, *E. coli* abundance was found in a steady state with the RA of 9.1%, as observed at day 28 (Figure 1).

### Pattern of bacterial family during cholera and recovery

**Figure 2:**
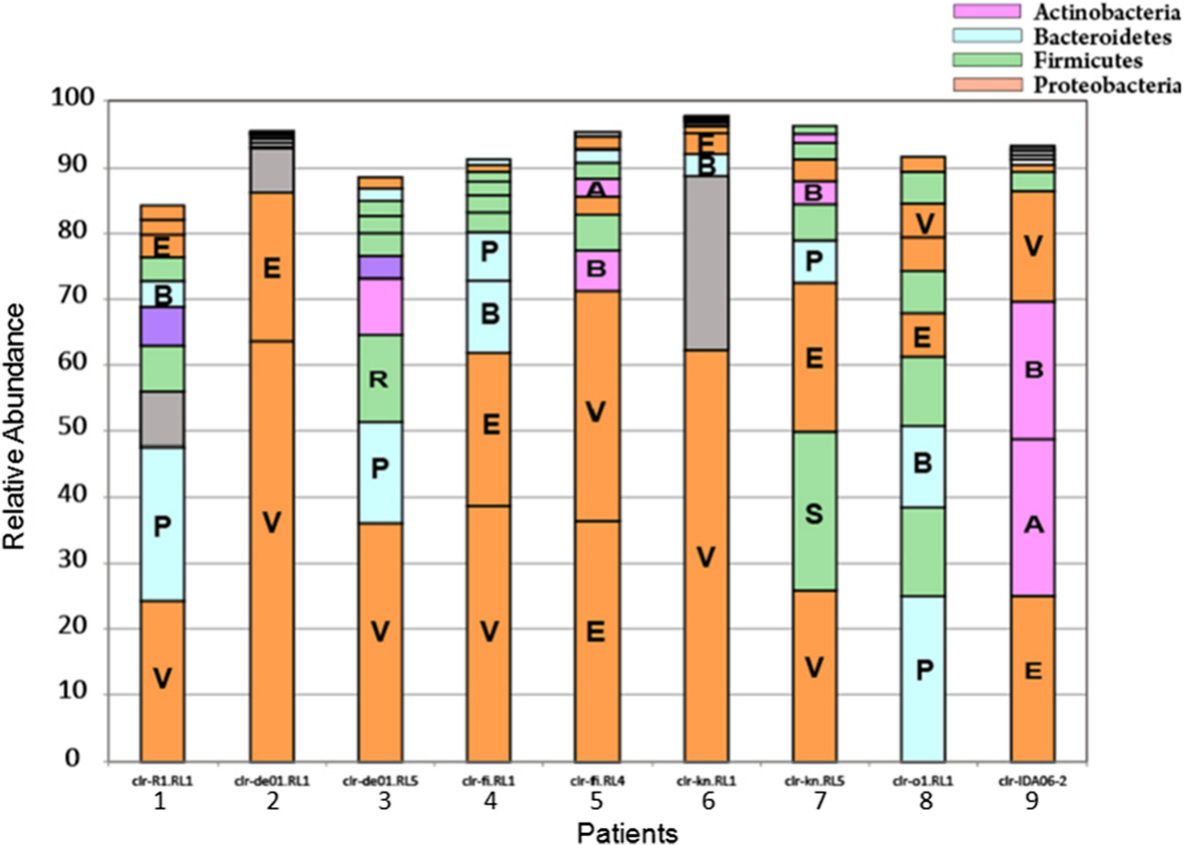
**Dominant bacterial groups in the gut of children with acute cholera at the taxonomic level of phylum.** Alphabets in the bar are described as follows: A, *Actinobacteriaceae*; B, *Bacteroidaceae*; E, *Enterobacteriaceae*; P, *Prevotellaceae*; R, *Ruminococcaceae*; S, *Streptococcaceae*; V, *Vibrionaceae*.

**Figure 3:**
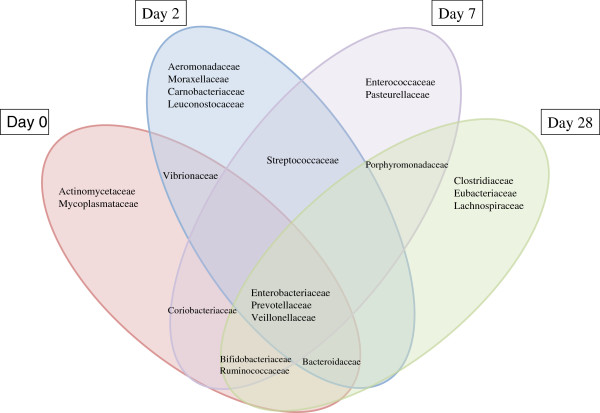
**Venn diagram showing the shift of commensal and pathogenic bacteria of the top ten bacterial families (by relative abundance) during recovery process in the gut of children with cholera.** Day 0, acute cholera; Day 2, antibiotics administrated; and Day 7 to Day 28, recovery period.

**Figure 4:**
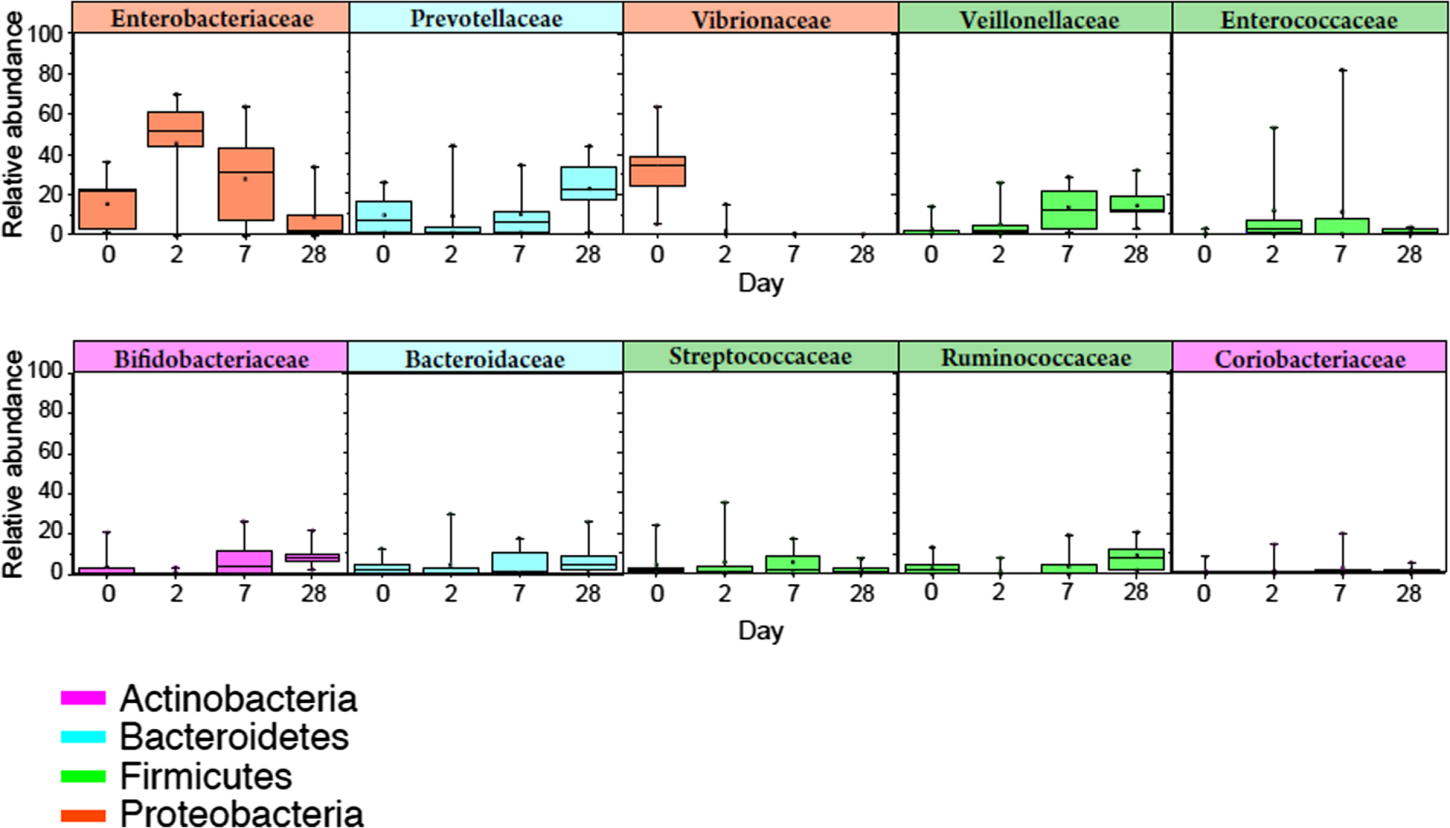
Comparison of relative abundance of dominant bacterial groups in children (n=9) with cholera on different time periods at the taxonomic level of family.

When top ten bacterial families in each of the children suffering from acute cholera (day 0) were determined, we observed significant inter individual variations of the dominant bacterial families (Figure 2), which accounted for more than 90% of the bacterial flora in nearly all of the children. *Vibrionaceae*, which was common in all nine children, had the highest relative abundance in six children, while *Enterobacteriaceae, and Prevotellaceae* were predominant in rest of the children (Figure 2). Although the pre antibiotic patterns of microbiota varied greatly for each of the children, bacteria belonging to the family *Enterobacteriaceae, Prevotellaceae, Actinomycetaceae, Mycoplasmataceae, Streptococcaceae,* and *Veillonellaceae* appeared to be the second most predominant in all nine children. The bacterial families observed at day 0 altered in the following days as there was selection pressure of antibiotics in the first three days of convalescence and in the first week (day 7) and fourth week (day 28) of recovery. It was remarkable that several different commensal and pathogenic bacteria that were identified at day 2, of antibiotic therapy, changed slowly as the children progressed towards recovery at day 7 and day 28 when the children returned home and were living with their families (Figure 3). For example, the observed relative abundance of some strictly anaerobic gut microbiota belonging to the family *Bacteroidaceae, Bifidobacteriaceae*, and *Ruminococcaceae* which was lower at day 0 compared to day 28, declined further as the antibiotic therapy began and increased again on the following days. In contrary, several other bacteria belonging to the family *Enterococcaceae,* and *Veillonellaceae* together with *Enterobacteriaceae*, to which *E. coli belongs,* increased several fold during antibiotic therapy period (day 2), although for unknown reason these differences were not consistent in all of the children suffering from cholera (Figure 4).

### Patterns of major bacterial phyla in cholera and during recovery

**Figure 5:**
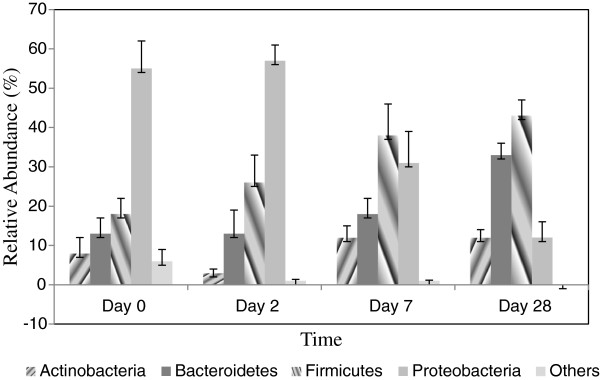
Relative abundance (percentage of sequences) of the dominant bacterial phyla in the gut of children with cholera on different time points.

Almost 98 – 99% of the sequences in all of the cholera children were found to belong to the four most populated bacterial phyla in human gut, namely Proteobacteria, Firmicutes, Bacteroidetes, and Actinobacteria. At day 0, the relative abundance (mean ± sem %) of Proteobacteria, Firmicutes, Bacteroidetes, and Actinobacteria was 55 ± 7, 18 ± 4, 13 ± 4, and 8 ± 4, respectively, in total fecal microbiota of all the nine children with cholera. These proportions did not change significantly before day 7 when an increase in number of all bacteria except Proteobacteria was evident. Subsequently, the relative abundance of the commensal gut bacteria belonging to Proteobacteria, Firmicutes, Bacteroidetes, and Actinobacteria changed (mean ± sem %) to 12 ± 4, 43 ± 4, 33 ± 3, and 12 ± 2 percent, respectively, at day 28. During cholera, gut bacteria of the strictly anaerobic phyla namely Firmicutes, Bacteroidetes, and Actinobacteria were reduced by 25%, 20%, and 4% respectively, while Proteobacteria was increased by 43% as compared to day 28. The increase of bacteria belong to Firmicutes and Bacteroidetes, and the decrease of Proteobacteria recorded at day 28 were statistically significant (p<0.05) (Figure 5).

### OTU analysis

Bacterial diversity was estimated on different time periods in cholera children (n=9) by enumerating the operational taxonomic unit (OTU) and we obtained (mean ± SD) 165 ± 71, 95 ± 72, 100 ± 32 and 157 ± 35 OTU at day 0, day 2, day 7 and day 28 respectively. OTU number was significantly reduced (*p* < 0.05) at day 2 and day 7 compared to day 0.

## Discussion

Gut microbiota play an important role in nutrition and healthy living of children. In this study, altered bowel movement particularly high purging in acute watery diarrhea due to cholera was found to be a basis for both qualitative and quantitative changes in the gut microbiota of affected children. Data presented in this study clearly showed that cholera partially washes out the gut resulting in loss of important commensal bacteria, which presumably creates room for harmful proteobacteria to colonize in disproportionately larger proportion and settle down in the gut, a scenario that we have observed in malnourished children of Bangladesh (Monira *et al*., 2011).

A wide variety of microorganisms including some human pathogens do not respond to artificial media when culturing methods are employed. Although non-culturable state has been established as a survival strategy, a great majority of intestinal microbiota are obligate anaerobic and extremely oxygen-sensitive in nature. Because of the complex nutritional requirements, recent estimates of *in vitro* culturability of bacteria were shown to range from 15 - 58% [10,11]. In the present study, we investigated bacterial community dynamics in fecal samples of children suffering from acute watery diarrhea (cholera) using culture-independent molecular (metagenomic) tools. Pyrosequencing and analyses of the amplified 16S rDNA allowed us to estimate the relative abundance of major bacterial community in gut. As previously demonstrated, this is a well-adapted method to detect pathogenic agents of diarrhea [12] as well as to determine the commensal gut microbiota in well nourished and malnourished children [13]. As observed in the present study, during acute phase of infection, *V. cholerae* was found to be the predominant species, followed by some other bacterial species, which were less abundant and may presumably be due to expulsion of the major gut microbiota resulted by purging in cholera. This appears to be in agreement with data presented by several culture-based studies (aerobic and anaerobic) showing the reductions of bacterial species in watery stools of severely purging adult patients with cholera [7,14].

It is now widely understood that the gut bacteria can influence the energy absorption from foods and the state of health [15,16]. Although information about strictly anaerobic (commensal) microbiota in the gut of children with acute watery diarrhea (cholera) is lacking, according to a recent quantitative PCR-based study [17], bacteria belonging to *Bacteroides-Prevotella-Porphyromonas* group, *Eubacterium rectale*, *L. acidophilus* and *F. prausnitzii* groups were low during diarrhea compared to recovery state. Moreover, *Bacteroides, Bifidobacterium, Lactobacillus*, and *Eubacterium* were shown to be less abundant in gut during diarrhea [9,18]. The data presented in this study demonstrate a clear picture of the gut bacterial communities in cholera and its recovery process, showing a decreasing trend of the bacteria belonging to the family *Bacteroidaceae, Prevotellaceae, Bifidobacteriaceae, Clostridiaceae, Faecalibacteriaceae, Megamonas, Megasphaera* etc., in children suffering from cholera. This is presumed to be due to the observed overgrowth of the invading pathogen, *V. cholerae*, and the resulted alteration of the strictly anaerobic environment that prevails in the normal human gut.

Cholera patients are routinely treated with a 1–3 day course of effective antibiotics [19,20] along with rehydration therapy, to shorten illness and reduce both rapid water loss [19,21] and the period of *V. choleare* excretion. However, antimicrobial agents often inhibit sensitive population causing shift in the intestinal microbial populations, with a decrease in anaerobic bacteria and a concomitant increase in aerobic bacteria [22,23]. Likewise, the relative abundance of four strictly anaerobic bacteria namely *Bacteroides, Clostridium, Faecalibacterium, and Bifidobacterium*, which were found in the gut of six children during acute cholera in the present study, reduced significantly during the period of antibiotic therapy (data not shown). Antibiotics namely, cephalosporins and some penicillins were shown to affect the gut microbiota and facilitate colonization of the gut by *Enterococcus* especially the ones that are resistant to vancomycin [24]. Although the antimicrobial susceptibility pattern of gut microbiota was not determined in the present study, an increase of the Enterococcus was evident during antibiotic receiving period when *E. coli* flourished rapidly and became the most abundant bacteria, suggesting the role of antibiotic in facilitating the resistant lineages of *E. coli* to selectively colonize the gut, as shown recently in pigs fed with performance-enhancing antibiotics [25]. The other possibility of this sudden increase of *E. coli* and *Enterococcus* at day 2 might be that the total bacterial number in the gut, including these two groups of bacteria, decreased during administration of antibiotics, but the abundance of *E. coli* and *Enterococcus* increased relatively because they were either less or not susceptible to those antibiotics than other gut bacteria.

We observed substantial inter individual variations of the bacterial genera in children with cholera from day 0 to the end of the study period. These children were randomly selected from those attending the outpatient department of the Dhaka Hospital and on the basis of inclusion criteria, acute watery diarrhea (cholera). They came from different localities and were consuming different diets. As diet and metabolism have a profound relation with indigenous bacterial populations [26-28], it is possible that these children might possess individual microbiota inventory related to the nature of diets. In our study we have observed that only three bacterial genera e.g., *Vibrio, Escherichia, and Streptococcu*s were common in all 9 children during acute cholera when complete data set was analyzed. Although *Escherichia and Streptococcus* were present among the gut bacterial community after one month, their relative abundance was very low compared to other bacteria namely, *Bifidobacterium, Veillonella, Faecalibacterium, Eubacterium, Prevotella, Ruminococcus, Clostridium, Enterococcus, and Megamonas,* which were the most abundant gut microbiota in all tested children at day 28. Now the question is, why the type and abundance of restored gut microbiota were so divergent among the hospitalized children, considering that they received similar care in terms of volumes of rehydration solution and type of semisolid and solid foods. Besides, the prolonged presence in trace amount of pathogenic *Vibrio* among the commensal bacteria of two children during the process of recovery at day 28 was interesting in the present study. Our study was limited up to 28 days, and so, it is not understood whether the pathogenic *Vibrio* in the two children eventually settled down as commensal among the gut bacteria. If this occurs, it is plausible to propose healthy human carriers for cholera and other pathogenic bacteria, although further study is needed to understand more about this. Nevertheless, the floral diversity observed in the post-cholera children in the present study suggests that the type of colonic flora may be determined by as yet unknown factor(s) that differ between individuals.

Since it is becoming increasingly evident that gut microbiota help prevent disease [29,30] and have profound influence on nutrition and health [31], the observed lower abundance of anaerobic bacteria in the gut of children during cholera and convalescence might have negative impact on post-cholera recovery process. This is presumable because each of the component bacteria of the gut community has their important role to play namely by digesting food that allows their host to optimally absorb nutrients and energy. Therefore, the observed lower abundance of major commensal bacteria during cholera and convalescence might affect the normal growth of children leading to growth stunting, considering that the bacterial diversity in the gut is positively linked with the status of health [13]. The results of the lower abundance of major commensal bacteria observed during cholera and convalescence in the present study appear in agreement with our previous study in which we observed that the concentration of short chain fatty acids (SCFAs), the metabolic products of gut bacterial fermentation, was significantly lower in children suffering from cholera [32]. The major limitation of the present study may be that, we worked with a small number of children with cholera. Nevertheless, the data presented in this study on metagenomes of the gut using high throughput pyrosequencing is the first of its kind, which provide a snapshot of the bacterial community in children during cholera and its recovery. While future study designed with a large cohort of cholera patients of different age groups remains an important area of interests, post-cholera intervention study to accelerate the restoration of commensal gut microbiota seems to be of great public health significance.

## Conclusion

Cholera infection appears to induce expulsion of major commensal bacteria belonging to the phyla e.g., Bacteroidetes, Firmicutes, and Actinobacteria, and facilitate harmful bacteria belonging to the phylum Proteobacteria to colonize the gut, as observed during acute and convalescence states. The observed floral disruption due to cholera might explain the wide-spread malnutrition in children of Bangladesh, where diarrheal diseases are endemic. Post-cholera intervention to promote the restoration of desired commensal can be of great public health significance.

## Materials and methods

### Study subjects

This study was conducted on nine children suffering from acute watery diarrhea in the International Centre for Diarrheal Disease Research, Bangladesh (icddr,b) and selected from our previous study [4]. The acute watery diarrhea was confirmed as cholera by dark field microscopy before enrolment in the study. The age of the children was between 2 and 3 year and they had history of acute watery diarrhea for less than 72 hours with moderate or severe dehydration. Their baseline characteristics are presented in Table 1.

**Table 1:** Baseline characteristics of cholera children included in the present study

**Criteria**	**Children with cholera** (n = 9) *
Age (month)	27.9 ± 4.8
Male/Female	4/5
History of diarrhea (hour) before admission to hospital	10.9 ± 7.1
*V. cholerae* positive by Dark-field microscopy	Yes
Antibiotic use	No antibiotic was given before reporting to hospital

*Values are presented as mean ± SD.

### Sample collection

Fecal samples were collected aseptically from each patient on day 0 (immediately after admission and before administration of any medication), day 2, day 7, and on day 28. The first three samples were collected by study nurse in the Dhaka Hospital, icddr,b and rest of the sample was collected from their home. Health workers visited the home of the study children the day before sampling and talked to their mothers, gave the stool collection containers and taught the collection procedure. Health workers collected the sample on the next day morning and maintaining cold chain brought to the laboratories. During their hospital staying, these children received oral rehydration solutions for maintenance of hydration until recovery from diarrhea. They also received the usual antibiotic therapy following standard protocol of Dhaka Hospital- parenteral ampicillin 100 mg/kg in four divided doses (6 hourly) plus Inj. gentamicin 5 mg/kg in two divided doses (12 hourly) for five days. All children with cholera also received erythromycin for cholera in a dose of 12.5 mg/kg per dose every six hours for three days. These children received normal hospital diet when they were able to take that. The ethical review committee of the icddr,b had approved both the clinical study and the present study, and informed consent was obtained from the parents or legal guardians of the child before enrollment. Also, the ethical review committee of the Research Institute for Microbial Disease, Osaka University, had approved the present study.

### Storage of sample

Samples were immediately preserved at −20°C freeze after coming to laboratory and stored until processing for DNA extraction.

### Extraction of total chromosomal DNA

DNA was extracted from fecal samples according to the method of Magne *et al.*[33]. 125 mg (wet weight) fecal sample was suspended in 625 μl breaking buffer [0.8 mol/L guanidinium isothiocyanate, 4% N-lauroyl sarcosine, 20 mmol/L Tris (pH 8.0), 80 mmol/L sodium phosphate buffer (pH 8.0)] and incubated for 1 hour at 70°C. Afterward, 750 μl glass beads 0.1mm in diameter (Sigma, St Louis, MO) and 15 mg polyvinylpolypyrrolidone were added. Bacterial cells were lysed in a vortex mixer at high speed (10 cycles consisting of 1 minute of vortexing and 1 minute of storage in ice). The mixture was centrifuged at 20,000 g for 3 minutes at 4°C. After recovery of the supernatant, the pellet was washed 3 times with 200 μl TENP (50mm Tris–HCl [pH 8.0], 20mm EDTA [pH 8.0], 100mm NaCl, and 1% [w/v] polyvinylpolypyrrolidone). The 4 obtained supernatants were pooled. Nucleic acids were extracted with one volume of phenol. The aqueous phase was washed twice by use of chloroform-isoamyl-alcohol (24:1). DNA was precipitated by use of 100% isopropanol, and the pellet was washed with 70% v/v isopropanol, dried, and resuspended in 50 to 100 μl of sterile water and stored at −20°C. The amount and integrity of DNA were estimated by use of 1% (w/v) agarose gel electrophoresis containing ethidium bromide (1 mg/ml) in 1 X TBE (Tris Borate EDTA).

### Universal primer PCR

Universal primer PCR was performed by following method, as described previously [34]. Precisely, PCR assay was performed using a primer set (784F: 5^′^-;AGGATTAGATACCCTGGTA-3^′^ and 1061R: 5^′^-CRRCACGAGCTGACGAC-3^′^). The primer set targets the V5-V6 region of the 16S rRNA genes. To amplify the targeted region, 1 μl of extracted DNA was served as the template in 50-μl reactions using Prime STAR HS premix (Takara Bio Inc., Japan). Each reaction mixture contained 10 pmol of each primer. The PCR conditions were 30 cycles of 98°C for 10 sec, 55°C for 15 sec and 72°C for 20 sec. Two 100 μl of 3-cycle reconditioning PCR reactions were performed per sample to eliminate heteroduplexes [35], with 10-μl aliquots of the initial PCR product mixture as the template and other PCR conditions unchanged. Products of the two reconditioning PCR reactions per sample were combined and purified using QIAquick PCR purification columns (Qiagen). The amplified PCR products were used as a template for pyrosequencing with the GS Junior platform (454 Life Sciences).

### 454 sequencing and data analysis

Relative abundance of bacterial phyla in fecal specimens was estimated by sequencing the PCR amplicons targeting the 16S rRNA gene for the DNA samples extracted from each fecal specimen. For this, pyrosequencing of the amplified 16S rDNAs was performed by following the manufacturer’s instruction using MID tags (Roche Diagonostics, Japan). Sequencing runs yielded 16,318 reads for one sample on the average. Sequence reads with low mean quality score below 30 and shorter length below 200 bp were filtered out by using BioRuby [36]. After filtering, the total numbers of remaining reads were 13,966 reads for one sample on the average. The average sequence length of the remaining reads was 289.7 ± 4.2 which corresponds closely to the length of amplicon product. These remaining reads were then subjected to a data analysis pipeline implemented in BioRuby [12]. The pipeline is composed of modified rRNA database “silva” release 94 and NCBI Taxonomy Database [37,38]. In the database silva, data records with ambiguous annotations for example soil bacterium or marine bacterium were excluded manually. Bacterial rRNA typing was performed by BLASTN search against the manually curated silva database using a threshold of E-value < 1E-40. The NCBI Taxonomy Database was locally reconstructed using MySQL. Taxonomic assignment of each hit of BLAST search was conducted by tracing data entries in the NCBI Taxonomy Database. The estimation of operational taxonomic unit (OTU) was performed by the program ESPRIT [39]. The OTU number at a genetic distance value of 0.10 was employed to avoid overestimation.

### Statistical analysis

Data were entered into a personal computer using a statistical package (SPSS version 11.5; LEAD Technologies Inc., Charlotte, NC, USA). Baseline characteristics of the study children were compared by analysis of variance. Differences between groups were analysed for significance using t-test. Data are presented as mean ± SD.

## Competing interests

The authors declared that they have no competing interest.

## Authors’ contributions

SM worked for sample collection, storage, DNA extraction, compilation of data, and manuscript writing. MA was involved in the overall study design, implementation, and manuscript writing. TI led SN, KG, and KI in pyrosequencing of genomic DNA and data analysis; TI also took part in critical review and editing of the manuscript. NHA assisted in sample collection and primary processing, SKIA in data analysis. HW was involved in study design and provided financial support to MA. TH, TN managed funding for pyrosequencing. All authors read and approved the final manuscript.
